# Temperament impact on eating disorder symptoms and habit formation: a novel model to inform treatment

**DOI:** 10.1186/s40337-024-00998-x

**Published:** 2024-03-19

**Authors:** Laura Hill

**Affiliations:** 1 Department of Psychiatry, University of California, San Diego, CA USA; 2 Adjunct Associate Clinical Professor of Psychiatry and Behavioral Health, The Ohio State University, Columbus, OH USA

**Keywords:** Eating disorders, Temperament, Symptoms, Habits, Traits, Model, Anorexia nervosa, Bulimia nervosa, Binge eating disorder, Neurocircuits

## Abstract

**Background:**

Temperament has long been described as the biological dimension of personality. Due to advancing brain-imaging technology, our understanding of temperament has deepened and transformed over the last 25 years. Temperament combines genetic, neurobiological and trait research. Temperament has been included peripherally in some eating disorder (ED) treatment approaches but has been ignored by most. Temperament fills a fundamental treatment gap by clarifying who is more vulnerable to develop ED and why some individuals are susceptible to specific ED symptoms while others are not. In addition, temperament targets possible treatment solutions.

**Main text:**

There is a need for a novel model that incorporates and explores the role of temperament in ED treatment intervention. This paper is a metaphoric temperament model to inform treatment intervention. It describes how temperament traits influences new decisions which impact new behavioural responses. In turn, it neurobiologically tracks how and why the brain efficiently transforms new decisions into new habits. This model integrates both temperament and habit research to explore (a) what temperament is; (b) how new decisions develop into habits neurobiologically; (c) that the brain wires destructive symptoms into habits in the *same* way that it wires healthy/productive behaviours into habits; (d) traits that trigger ED symptoms are the *same* traits that influence productive behaviours; and in regard to treatment implications (e) when treatment structure and intervention target client temperaments, the potential for new healthy “trait-syntonic” habits could develop.

**Conclusions:**

This paper introduces a metaphoric model that synthesizes and integrates temperament neurobiological and trait findings with ED symptoms, habits, and client trait-based solutions. The model synthesizes and integrates different research domains to establish a brain-based foundation to inform treatment intervention. The model targets clients’ temperament traits as central collections of innate self-expressions that could be utilized as tools to redirect client trait-syntonic ED responses into trait-syntonic productive outcomes. The brain bases of temperament and habit formation serve as a biological foundation for ED treatment intervention.

## Abstract (plain language summary)

Temperament is the biological, or brain-basis of our personality. It defines who we are and who we are not. Temperament influences how each person thinks, feels, and responds differently. Traits are one’s natural personality expressions. For example a person may have natural tendencies, or traits, to be extroverted and determined. A person could express the same traits differently ranging from healthy/productive through unhealthy/destructive responses. Research has found that some traits, when expressed in destructive ways, influence the development of eating disorder symptoms. Some of those traits include perfectionism, impulsivity, and anxiety. However, these very same traits could be expressed in productive ways. In essence, traits *are* the biological triggers beneath one’s responses and set the stage for productive to destructive habitual behaviours. Persons cannot eliminate their traits, but they can express them differently. Treatment could capitalize on client traits to help clients explore productive responses instead of turning to destructive habitual symptoms. Productive habits that are aligned with one’s traits could more naturally persist and form long-term habits. Working *with* a client’s temperament, verses overlooking it, holds hope for habitual productive change to replace ED symptoms.

## Introduction

### Background and introduction to a novel temperament eating disorder model

Temperament has long been described as the biological dimension of personality [1]. Core elements of temperament include genes, neurocircuit networks, and traits [2–4]. Temperament traits are internal qualities, or personality expressions that we cannot see and yet influence how each of us thinks, feels, and responds differently. Temperament biologically defines who we are and who we are not. Due to advancing brain-imaging technology our understanding of temperament has deepened and transformed over the last 25 years [5]. Temperament researchers are now able to explore how genes influence neurobiological networks that translate into and impact trait expressions [6–9]. Trait-influenced thoughts transform into trait-influenced actions. How we act, what we do, and potentially when we do something are all triggered in part by traits. While many traits influence healthy daily responses, research has found that some traits appear to increase vulnerability to develop eating disorders (ED) [10–13].

Researchers in the field of ED are expressing increasing concern regarding the limited diagnostic focus on ED behavioural symptoms and advocate for an expansion in our way of thinking about this severe range of illnesses [14]. Novel ED models are needed that reflect current research. This paper describes a metaphoric model that integrates ED research on temperament and habit formation. It describes how the brain neurobiologically integrates input to make new decisions. It tracks the process of how the brain starts with new decisions and efficiently rewires decisions into habitual actions. This brain-based model provides a framework out of which treatment interventions could be shaped to augment ongoing ED therapies.

Overall this temperament model explores and illustrates: (a) what temperament is; (b) how new decisions develop into habitual actions neurobiologically; (c) that the brain wires destructive symptoms into habits in the *same* way that it wires healthy/productive behaviours into habits; (d) traits that trigger ED symptoms are the *same* traits that influence productive behaviours; and in regard to treatment implications (e) when treatment structure and intervention target client temperaments, the potential for new healthy “trait-syntonic” habits are more likely to develop. This temperament and habit model fills a gap in ED research by identifying and addressing underlying temperament traits and brain responses that impact outward ED behavioural symptoms. The model does not emphasize interpersonal and environmental factors that shape temperament since they are covered in other models [15, 16].

This model expands on the temperament model described in *Temperament Based Therapy with Support for Anorexia Nervosa* by Hill et al. by tracking the neurobiological process from new decisions to habit formation, and describes how traits “color” this process and impact long-term change [17]. In addition this model provides a framework for a range of ED, not solely anorexia nervosa (AN). Recognizing how traits impact the formulation of new decisions and habits serves as a guide to structure a temperament-based treatment intervention.

Over the last few decades, research and treatment providers have approached temperament traits as constructs. Nobel prize winner for physiology, Eric Kandel stated that what was once thought of as constructs, such as temperament traits, can now be viewed in terms of genetic expressions that take shape through neural circuits [2]. This paradigm shift allows us to fundamentally alter the way we view ourselves and our clients. People are not blank slates, as J. Watson wrote in the 1930’s, upon which externally-determined changes can be imposed to produce any outcome that clinicians determine are appropriate [18]. Temperament is unique for each person. It both limits and frees a person to express themselves.

Eating disorder clinicians could benefit by better understanding temperament because temperament plays a central role in determining who develops anorexia nervosa and other ED [3]. Eating disorder-related traits go beyond the trait models described in Zuckerman’s Alternative Five or Eysenck’s Big Three system, and Costa and McCrae’s Big Five [19]. Traits identified as common among ED include perfectionism, harm avoidance, impulsivity, and obsessive traits [20–23]. Recognizing a client’s unique temperament profile fills a treatment gap in explaining why some persons develop specific ED symptoms, such as acute food restriction, while others develop different or overlapping ED symptoms, such as those with AN who also binge and purge.

## Main text

### What is temperament?

Temperament begins biologically through the genes one inherits. The DNA of one’s genome determines each person’s traits. The genome formulates how the fetal brain structures its neural circuits to establish a wide range of trait expressions [24]. Our traits are with us for life. However, they are continually shaped by environmental influences. Once traits are genetically coded, environmental influences begin to impact trait expression during fetal development and continues during infancy, childhood and throughout one’s adult life.

Overall, both biological and environmental processes establish one’s personality. Following Cloninger’s personality model, temperament has been viewed as the biological or nature side of personality, and character has been described as the nurture side that shapes a person’s responses through environmental influences [17, 25]. Currently researchers describe these two entities as intertwined, with temperament continuously affecting character and vice versa [26]. Temperament traits continuously trigger responses from the inside while the environment shapes a person’s character from the outside. Extreme environmental stimuli significantly impact behavioural responses, such as times of trauma and other adverse events. Trauma can significantly alters one’s character. Acute starvation, severe binge eating and purging episodes are ED traumatic examples that could cause epigenetic changes [27–30]. This means that the environment and one’s behaviours impact neurobiological and trait responses through alterations in gene expressions [29, 31, 32]. Gene expressions can be turned “on or off” throughout life based on influences from e.g., biological, dietary, environmental, and interpersonal experiences.

For example the environmental pressures to be thinner coupled with specific traits such as perfectionism, anxiety, and obsessiveness increase vulnerability to develop extreme dieting patterns that trigger gene expressions to alter [31, 33, 34]. However, the actual genes that code the identified traits remain constant. The environment can shape trait expressions, but it cannot change one’s traits. An example from nature is an oak tree. It is genetically coded to be an oak. That cannot change. It cannot become a maple or pine tree. But the way the tree grows, the extent of its branches, the height and breadth are all shaped in part by the environment. It remains that traits are the precursors to how a person responds, whether in calm to extreme environmental situations. When treatment focuses solely on ED symptoms and trauma, a major part of the problem and solution is ignored, one’s traits.

### The relationships among traits, symptoms, and habit formation

Temperament consists of the biological ingredients that define us and is neurobiologically expressed through our traits [22, 35]. Traits influence actions. Symptoms are approached therapeutically as problematic or destructive responses or actions [36–38]. However, this does not mean that a person simply chooses to do destructive actions. Research has found that traits influence the development of ED symptoms [22, 39]. Symptoms are in part outward actions triggered in part by internal traits, which are in turn influenced by abnormal neural circuits that fire differently from those who do not have ED [40]. Externally, ED symptoms include excessive food restriction, binge eating, or purging behaviours [34, 41, 42]. Internally, ED symptoms look like significant dysfunctional neural circuits within the brain.

Habits occur when actions, healthy or unhealthy, become automatic through repetition [43]. From a neurobiological perspective, habits are unconscious learned responses established through repetition [2]. They are predisposed by a person’s temperament [19, 44]. Habits are neurobiologically efficient ways of expressing one’s temperament and coping with environmental stimuli [2]. There are compelling behavioural and neural data that suggests habitual responses may underlie the persistence of ED, such as those with severe-and-enduring anorexia nervosa (SE-AN) [45, 46]. Eating disorder habits become stronger the longer the illness persists [47]. Overall, traits influence which habits develop. Treatment can help shape whether habitual actions remain symptomatic or become healthy/productive. Traits, symptoms, and habits are important targets in clinical treatment because one’s traits biologically influence which actions that one expresses productively to destructively.

**Fig. 1:**
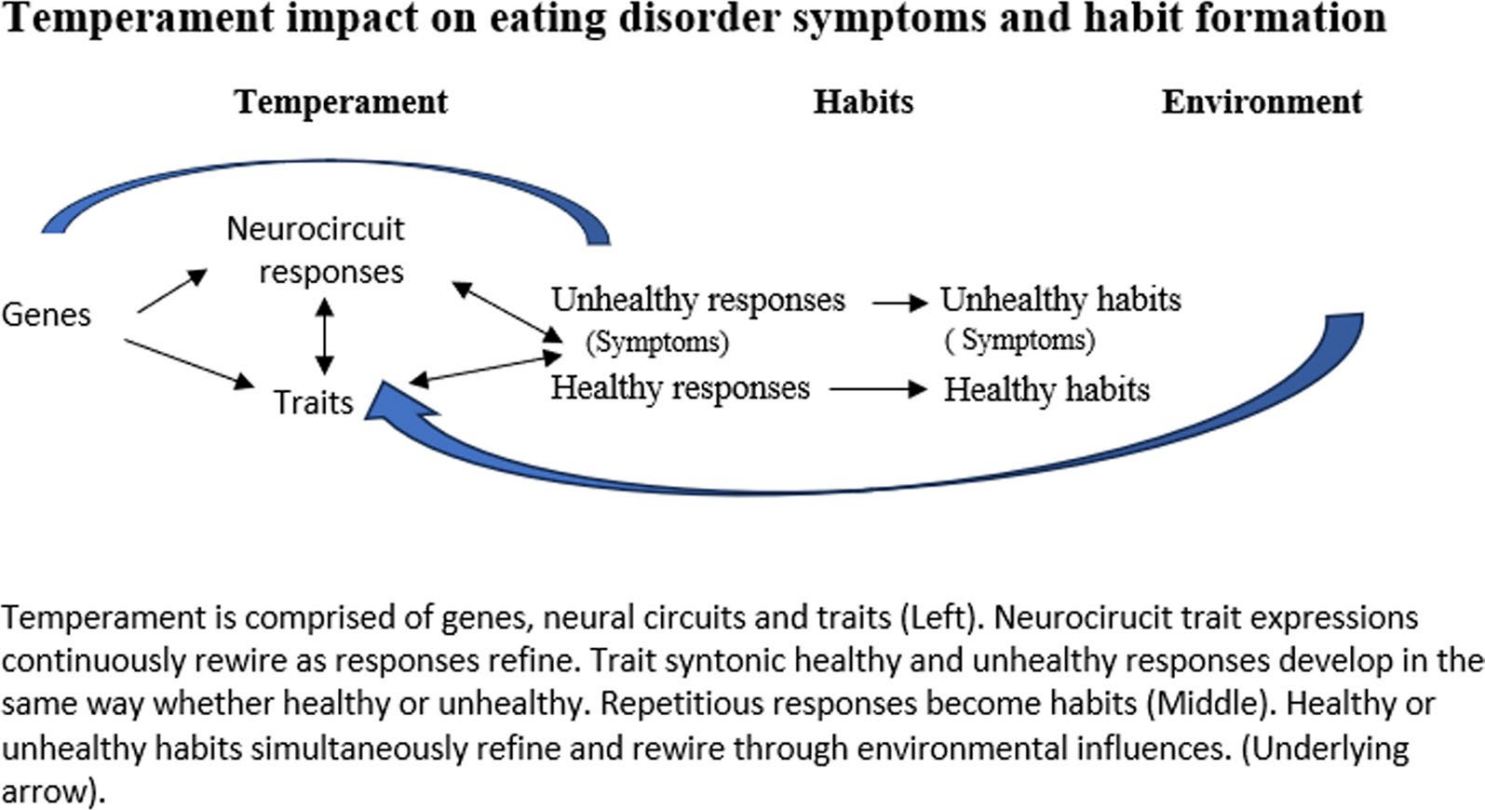
Temperament impact on eating disorder symptoms and habit formation

Figure 1 illustrates the relationships among temperament, symptoms, and habit formation. It visually describes that genes identify or code neural structures to develop trait expressions. Neural circuits continuously initiate and influence trait expressions and vice versa. The interactive process impacts trait expressions via neural circuit alterations which, in turn, impact habit formation. Environmental stimuli have an ongoing role in shaping trait expressions to become productive to destructive. Habitual behaviours strengthen brain circuit responses which in essence strengthens habits [48]. The paradox is that while genes and traits remain constant throughout life, trait expressions and their neurobiological rewiring continuously change. Clinicians could enhance their ongoing therapies with temperament-based, structured interventions that could help clients rewire their trait-syntonic ED symptoms into trait-syntonic productive habits.

Preventatively, the same traits expressed destructively through ED symptoms could be rerouted to be trait-aligned or trait-syntonic productive actions contributing to the persistence of productive habits. Productive habits become stronger the longer they are practiced in the same way ED behaviours become persistent [49]. Early childhood is a prime time to identify and structure trait expressions in productive ways. One child may be fearless, while another holds back and is shy. A preventative intervention could include teaching parents how to identify their child’s traits and offering tools to structure and shape opportunities for their child to express their traits as strengths. This could encourage the child to be more comfortable with themselves as they play, learn, and interact with others.

A preventative example could be a child who has high achieving, perfectionistic, competitive, and detailed traits. These traits are associated with both highly impactful accomplishments and also with AN. The parents could explore if the child naturally enjoys and has a propensity to focus on words. They could help their child enter a local spelling bee. The attention to detail, the need to compete and to get every word spelled correctly reroutes a potential to focus from counting calories to letters. Letters could be less burdened with social pressures around body size and ED symptoms. A spelling bee provides structure for attention and actions to focus on new words each day with friends and family. Upon winning the local spelling bee, the child may have the drive to achieve more by going to the regional spelling bee. The child’s traits become strengthened and habitualized productively.

Traits are considered healthy until problems begin to occur biologically, interpersonally, or psychologically. Could the obsessive tendency to spell each word correctly coupled with high achievement and perfectionism traits become problematic? Yes. What begins as a fun challenge with traits expressed within healthy limits for the child’s developmental abilities, could become a dreadful experience when problems develop. The same traits that influence the best in the child’s performance could also bring out the worst if actions become too extreme. Warning signs such as increased isolation from friends and acute anxiety when failing to spell each word perfectly may arise. Treatment providers could help parents identify external structures that align with the child’s traits and within the child’s tolerance levels.

Hypothetically, rerouting anxiety and obsessions could mean that a plan that the regional spelling bee is enough for achievement this year. Then the parents and teachers could help the child refocus interests onto a new project to give the same traits a new outlet of expression within healthy limits. The project could have criteria that the child temperamentally relates to, such as a science project that requires attention to detail. Ongoing projects provide opportunities for the child’s traits to be expressed in fun and productive ways. They are non-food and dietary focused to reroute and reduce the potential for the child to develop problems with calories and body size. This does not guarantee that an ED will not develop. It offers a trait-based reroute to prevent the possibility from triggering a cascade of biologically harmful symptoms, such as ED. Shifting trait expressions to more productive and healthier levels of expression is referred to as sublimation. Overall, the child could experience opportunities that celebrate their traits as strengths.

### How new decisions develop into productive or ED destructive responses/symptoms

Temperament is the neurobiological platform that defines and refines who a person is [50–52]. Overall, the brain is a global city containing millions of neural superhighway networks. They have intersections of communication that manage every aspect of the body in relation to environmental stimuli. While the brain remains the least understood of all body organs, advancing technology is zooming in with greater detail to observe what pathways are firing and how accurately or abnormally they are transmitting signals throughout the brain among those with ED [36].

New decisions are required when facing new interoceptive (internal), and environmental (external) experiences. The brain needs to assess and identify a new response. For example, “What do you want to do tonight?” is a new stimulus that requires a new decision and behavioural response. The brain directs cognitive attention to the new experience. The brain sorts through expansive amounts of neural data received from the physical senses and environmental stimuli. It also incorporates internal sensations, thoughts, and feelings. A high level of accuracy of the vast number of neurocircuit responses is fundamental in order for a person to make a well-informed decision.

How circuits respond to situations moment by moment contributes to one’s health as well as one’s ED. Brain research has compared neural responses of persons with ED, with those who have recovered from ED, and with those who have never had ED [53–55]. Findings have identified brain circuits that fire differently from those who do not have ED during and after the remission of ED [33, 54, 56, 57]. These findings indicate that some traits appear to increase the risk of developing and maintaining ED. For example, abnormal insular function appears to contribute to the inability to process gustatory, satiety and other interoceptive sensations causing errors in decision making [58–60]. Eating disorders in part consist of an overabundance of abnormally firing neurocircuits that contribute to habitual symptoms and relapses among those with AN and other ED [61–63].

If some to all of a person’s neural circuit responses are abnormally firing, judgement is skewed and clarity in decision-making is impaired. A simple parallel is one’s sight. If an individual is farsighted, it means that one’s eyes see objects clearly that are far away while they experience problems, or blurred vision, when seeing objects that are close. The blurring is caused by refraction errors in the eye influenced by genetic and environmental factors [64]. Decision-making involves a wide variety of neural circuits throughout the brain, with vision being but one area. Accurate neural circuit responses from one’s five senses and interoceptive cues are necessary to provide accurate neural information to navigate “clearly” through new personal and interpersonal decisions. In addition to trait influences, overall accuracy in neurocircuit responses is impacted when poorly supplied or well supplied with energy/glucose [65, 66]. Regardless, the brain makes decisions based on the neural signals it has at any given moment in time, accurate or inaccurate.

**Fig. 2:**
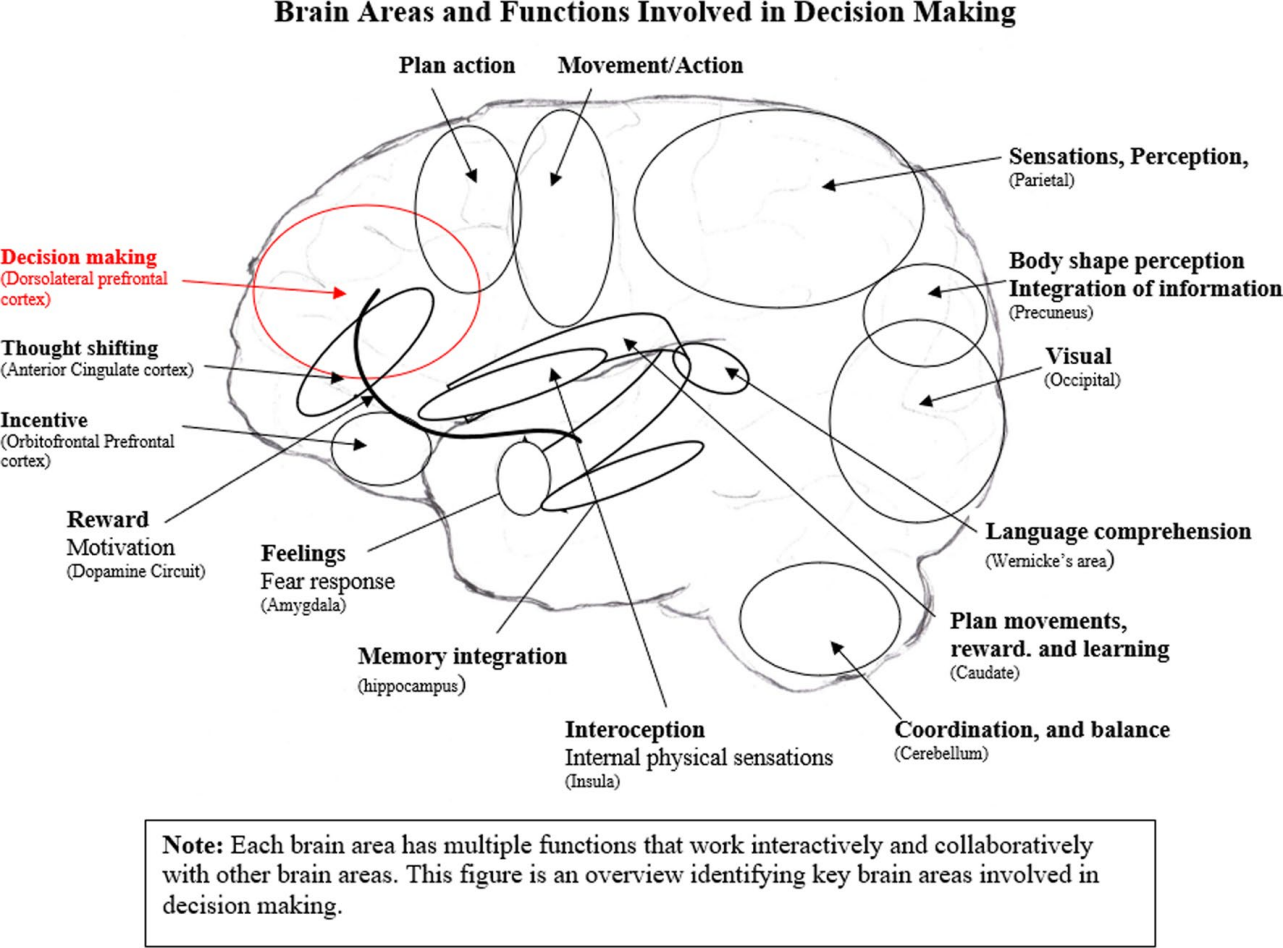
Brain areas and functions involved in decision making

Figure 2 identifies the home base, or executive site, where decisions are formulated. It is located at the front of the brain, the dorsolateral prefrontal cortex (DLPFC). This cognitive brain area draws upon a vast amount of neural circuit input to make decisions regarding what to do or not to do in novel experiences. If neural circuits inform the DLPFC with accurate information, the neural information consolidates with clear information to make accurate decisions. If one or more of the neural circuit pathways from various brain areas offer erroneous cues, then thoughts, feelings, perceptions, and sensations tend to “blur” decisions [67]. Behavioural ED symptoms are, in part, consequences of trait-based abnormal neural circuit input that “blur” decisions [33, 38, 54, 68, 69].

Figure 2 demonstrates the large number of brain areas and massive number of neurocircuit networks required throughout the brain each time a new decision is made. It is overwhelming to comprehend. Yet the brain incorporates the immense neural data moment by moment every day. Overall, the more accurate the neural responses, the greater the potential for clear, productive responses, and vice versa. Decisions are skewed when input consists of a wide range of abnormal neurocircuit responses.

Neuroscience has identified differences in neurocircuit responses among those with ED compared to those without ED, in areas related to anxiety, interoception, and reward/punishment. This implicates neurocircuit involvement in symptom expression [33]. These neural alterations culminate at the DLPFC, skewing, and in some cases “blinding” decision-making for those with ED [69]. ED neurocircuit dysfunction includes errors in reward circuits that impact motivation and habit formation [38, 70, 71]. These dopamine neurocircuit responses are significantly lower in AN causing decreased motivation [72, 73], and vacillate between significantly low and excessively high dopamine responses among those with bulimia nervosa and binge eating disorder. This extreme vacillation increases one’s desire to eat significantly more compared to healthy controls [27, 74, 75]. Errors in interoception, one’s ability to experience physical sensations such as taste, satiety, hunger, or pain, highly impact decision-making [76, 77]. For example, a client cannot make an accurate decision to eat or to stop eating if they are unable to correctly assess if they are hungry or full. This does not appear to be due to one’s awareness or degree of mindfulness, but due in part to abnormal insular neurocircuit responses [78, 79].

In addition, there are neurocircuit errors in body image perception that contribute to body shape disturbances [79]. Neurocircuit errors in anticipation [80], contribute to increased anxiety [78, 81, 82], and to prediction errors among those with ED [70, 83]. Cognitive neurocircuit abnormal responses could contribute to increased inflexibility in decision-making [84, 85]. Reduced neural myelin across many neurocircuits, from reduced food intake, contributes to inflexible and rigid decisions [72, 86, 87]. Neural responses clarify if it is safe to respond or not to respond to new stimuli. Learned abnormal responses from former experiences continue to fold into current abnormal circuit responses compounding the repetitious development of symptomatic behaviours [38].

Dysfunctional neural circuit responses establish an increased inability to trust new decisions for those with AN [17]. Open-ended questions such as, “What do you want to do?” tend to trigger anxiety for those with AN, compared to persons without ED [46]. Open-ended questions imply unending options. For those with AN, this creates a problem in one’s expectation to sort through endless options to make a decision. Input from decreased insular and reward neurocircuits blurs input to the DLPFC causing doubt in decision-making. Offering two or three options recognizes the underlying inability to decide. Limited choices help the client compensate for skewed neural input and reason through each option to better trust their decision and reduce anxiety [17]. Is deliberate cognitive reasoning or over-thinking an act of “over control” as social media, families and some therapies indicate [16]? Or might it be the need to cognitively compensate by thinking intentionally about each option due to aberrant neural input transmitted to the DLPFC? This model points to the later.

Taken together, ED behavioural symptoms are triggered by a plethora of abnormal neurocircuit responses. Decisions are made based on skewed neural input. An assessment is made by the DLPFC drawing upon past and present neural circuit calculations and miscalculations [88]. The decision is transmitted, accurate or inaccurate, to the motor cortex areas to plan a new action for a response. The new action may range from productive to destructive depending upon neural and trait input and environmental influences.

### The brain wires destructive symptoms into habits in the same way that it wires productive behaviours into habits

What does the brain look like neurobiologically when habits form? While it is evident that behavioural habituation is caused by different mechanisms depending on the time frame when the new responses are initiated, the sensory pathways involved, and the hierarchical level of signal processing, the brain remains highly conservative in reducing habitual responses for the sake of survival. [89]. The brain efficiently shifts neural responses from multiple brain areas needed when making decisions, illustrated in Fig. 2, to the central area of the brain [90–92]. This area in part transmits the refinement of habitual actions. As habits form, roles in the brain shift.

Cognition, attention, planning, and reward responses, which hold primary roles in decision-making, shift to secondary and even tertiary roles as habits form. The amount of input needed from each brain area appears to depend on fine-tuning factors such as the novelty of circumstances each time the habitual action is repeated [91, 93]. The prefrontal brain areas have already assessed if the action offers reward, satisfaction, or reduced punishment. One habitual action may develop in response to abnormal reward cues and another due to abnormal interoception, or both. Nevertheless, the brain uses the *same* method to form habits, productive to destructive.

Habits are the brain’s method of using its energy efficiently, pruning synapses and reducing circuits when repetitiously responding to similar stimuli [2]. If the action is repeated, the brain shifts habitual responses to the central area of the brain [49, 59, 71, 94]. Habits continue to be informed by prefrontal, parietal and other brain areas, establishing a balance that is in constant flux as habits are refined [95]. Habits become efficiently simplified when repeated over time. Habitual neural responses also become stronger the more habitual actions are practiced aiding in the chronicity experienced in clients with severe and enduring eating disorders (SEED) [47]. As habits occupy space in the central area the brain, the prefrontal cortex is freer to respond to new stimuli to make new decisions moment by moment. With less neurocircuitry required for habitual actions, less energy is needed to express a habit than to form one. At a starved state, the brain might rely on habits such as purging or restriction to cope instead of facing new decisions [96].

Just as traits are not stagnant, neither are habits. Even when habitual actions are formed, they can be changed. Clinicians could target the “malleable moment” when a client enters into a *new* experience. It opens the opportunity for a *new* decision that requires a *new* action. Theoretically, each time a client makes a new decision, a temperament-based intervention could guide the client to explore productive, trait-syntonic actions.

What might this look like in treatment? This question ignites the following question. What if simple actions, the end-stage of decisions that form into habits, becomes the first stage in the decision making process? This is parallel with how the brain responds when faced with trauma. Action first, then think through the response. The brain temporarily side-steps cognition by reacting in three primary ways, flight, fight, or freeze. Traits automatically influence which response is taken. A temperament-based treatment could create interventions that replicate neurobiological ED responses in safe distressful through playful scenarios requiring the client to take action in the moment to solve a given problem. The clinical activities become the stage for decisions to play out.

For example, a novel therapy called Temperament Based Therapy with Support (TBT-S) has been developed to augment other therapies by identifying and helping clients reshape their trait expressions from destructive to more productive responses. It applies and integrates ED neurobiological and trait research with temperament-based clinical applications [17]. It requires the involvement of persons with whom the adult client identifies that they can turn to for support. These people are called “support persons.” During treatment sessions, child/adolescent and adult clients with ED, and their support persons are presented with current neurobiological research findings. The client is asked to self-assess how the research findings are similar to or different from their own experiences regarding reward and motivation, interoception, and decision-making.

The clients realize that, like farsightedness, they needs tools that are metaphoric glasses to compensate for “blurred” neurocircuit responses that impact their decisions. However, unlike the opportunity to be prescribed glasses to compensate for visual aberrations, outside of medications, there are no compensatory accessible methods to assess and correct for ED neural circuit responses. This leaves clients to resort to their trait-syntonic responses, such as binge eating, purging or excessive exercising to cope. However, these actions are destructive.

TBT-S offers structured experiential activities for clients with ED to solve problems utilizing action for clients to explore trait-syntonic solutions. In essence, decisions are made backwards for clients who have trouble trusting their decisions. Action first then cognition. One example is called “The Landmine” [17, 97]. The game is structured to provide the client with a safe framework to explore and identify how to compensate for their self-identified destructive traits and abnormal circuit responses.

The TBT-S intervention activity is too extensive to describe in this paper. In short, it creates a stage that parallels the client’s neurobiological and trait-based responses that have resulted in ED symptoms. The experience replicates the obsessive “noise” the client experiences as anxiety increases when faced with new decisions. It creates triggers (landmines) that replicate vulnerable moments when faced with new food intake. The game instructs the client to maneuver around the landmines “blind.” This is to parallel neural circuit misfiring, “blinding” their ability to trust their decisions. How the client solves the game and which traits the client draws upon to win, is played out in the process of getting through the landmines. After the client wins, (e.g., eats a new meal), they translates what actions they used and how into applications when faced with new decisions daily. The trait-syntonic responses utilized to win hold a key to become repetitive productive habits.

The client actively experiences what is needed to “reroute” from destructive actions to healthier actions. For example, adult clients consistently report, after failing many attempts to walk the landmine on their own, that they need “support” to help them get through new decisions. How to ask for support and what they want the supports to do is figured out during their actions in the game. The client instructs the support person on what they need them to do to help them through the landmines. When the client steps on a landmine, they consistently discover that their instructions to the support person need to be clearer and more defined regarding what helps and what does not help. The client enters a malleable moment when new required actions are placed before them to win the game.

At the end of the game, it is explained that their decision to draw upon a support person is parallel to reaching for one’s glasses for assistance to see more clearly. The parallel is also drawn between how the client and support person solved the problem to facing new decisions in everyday new situations [98]. Each client solves The Landmine differently, utilizing their own traits to influence how to utilize support to win the game.

The client reviews which traits they used to win the game. Trait-syntonic actions are processed regarding their responses to compensate for their “blurred’ decisions” in daily life. This helps clients to realize how the same traits are part of their own healthy solutions, not merely triggers to their ED behaviors. The goal is to replace ED symptom responses with productive trait-syntonic responses. Taken together, what to do and how to compensate for difficulty in making new decisions is presented through structured activities that encourage action-based experimentation for the client to discover their own trait-based solutions [17].

Just as the brain uses repetition to establish habits, so too could treatment intervention. After solutions are identified by the client through trial and error experimental actions, the client is given the opportunity to practice their self-identified new solution during subsequent treatment days or outpatient sessions, and at home. This repetition increases the probability for clients to develop new healthy habits. Healthy solutions are tailored by each client, which could develop into new habits.

This reverse treatment method means that cognitive and affective processing occur after the action. The therapeutic process becomes the structured stage to practice productive, trait-syntonic habits established by facing new decisions actively during treatment sessions. The client is less likely to practice at home if they have not already begun to actively practice new solutions during the treatment sessions. This process jump-starts cognitively exploring many options first and moves into action to see what “fits” first. After trait-influenced actions are identified, the decisions of how and when to apply the action becomes easier to identify. Once identified, new productive trait-syntonic productive actions could theoretically compete with and potentially replace a trait-syntonic symptomatic habit.

Practice, the act of doing the behaviour repeatedly, is key to new habit formation [2]. Symptom reduction may not be required to “reverse” a current symptomatic habit. Transformation of trait expressions could be achieved by introducing new trait-syntonic productive actions while symptomatic habits remain active. Repetition of new actions allows the brain to rewire new responses into new habits that could compete with symptomatic habits. Over time, the habits practiced more will strengthen and the less-practiced habits will weaken in neural strength. Old habits could fade away while the client gains new strength drawing upon trait-syntonic productive habits.

How might habit formation relate with SE-AN and SEED? For many with SEED, ED symptoms have endured for years or decades, not months. The neural circuits in the central brain area become stronger and expanded as new habits form in and around ongoing ED symptomatic habits. Additional habits that precede and are subsequent to ED symptomatic habits become strengthened and solidify, establishing neurobiologically strengthened additional rituals. As one habit leads to the next sequential habit, the breadth of habitual symptomatic rituals form, aiding in the persistence of the illness [44]. If environmental stimuli remain constant, ED habitual responses solidify.

Metaphorically, habits that precede and are subsequent to ED symptomatic habits become reinforcing neural suburbs around the city of symptom responses. The neural circuits expressing one symptom become enmeshed with circuits of additional symptoms. One neural network ignites the next expanding city-wide neural boundaries that dominate choices and new decisions. If the client is left to change alone, new productive responses could not penetrate the massive network of the habitual symptomatic neural city. Clients with SE-AN appear to resist traveling beyond their own neural city of habitual responses. External structure with the inclusion of support persons are needed to navigate into unknown avenues of change for those with SEED.

### Traits that trigger ED symptoms are the same traits that influence productive behaviours

Each person may inherit hundreds of personality traits designed by thousands of genes [12, 13]. Traits are not located in specific areas of the brain. Traits are expressed through neural circuit responses in various neural networks. Temperament traits biologically influence which actions are taken and which habits develop.

Traits influence one person to have tendencies toward impulsivity while another person may tend to be avoidant or to hold back. Some may inherit both traits. Individuals may also choose to respond outside of their temperament tendencies. This requires more focus, more intention, more concentration, and more energy than responding within one’s temperamental tendencies. Non-trait aligned actions are not as natural or easy to express as trait-syntonic responses. They require neural responses outside of one’s temperament-based neural patterns. Non-trait aligned actions could become habitual just like trait-syntonic actions. However, theoretically, the responses may take more effort and could more easily fade away over time than trait-syntonic habits.

**Fig. 3:**
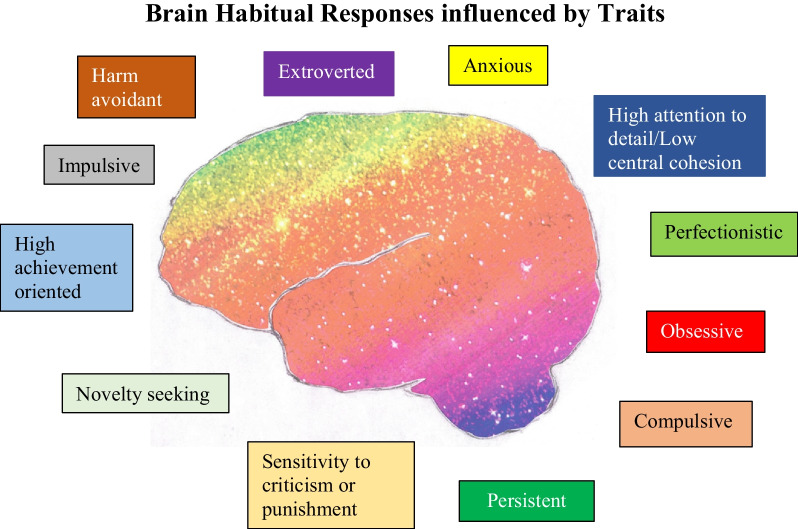
Brain habitual responses influenced by traits

Figure 3 in this temperament model is an image of the brain overlaid with a wide range of multiple colors. The colors and their values serve as a metaphor for multiple ED traits that have wide ranges of expressions from healthy/productive to unhealthy/destructive. The colors are not literal and they do not reside in specific areas of the brain. Figure 3 identifies common ED traits [99]. Traits influence the direction of new decisions. Traits “color” or influence natural tendencies for habitual neural connections. Overall, repetition of trait-syntonic behaviours is more natural compared to non-syntonic-trait responses. One may wonder if the brain has to unlearn, or reverse one’s habit, to shed an unhealthy/destructive habit [45]. This model theorizes that new trait-syntonic habits could be learned and compete with, and potentially replace, unhealthy habits.

If a client tries to adopt behavioural responses outside of their temperament, the responses may not “fit well” requiring more concentration and intention to be sustained. This may be one reason why many clients who try to apply behavioural changes during treatment that are not syntonic with their own traits may relapse to their trait-syntonic behavioural symptoms after treatment. If clinicians do not help clients identify their own traits, clients may try to adopt what is defined as appropriate actions, but they don’t “fit well” for the client. If clients are not given opportunities to actively explore how to respond productively and congruently within their own temperament, then they may find themselves feeling inept or deficient when their new actions are not as easy for them as they appear to be for others. This could increase anxiety, guilt, and stress.

An example of treating outside of a client’s traits is an adult client who dedicates attention, time, and effort to respond to a program treatment goal that directs the client to eat a different meal every day. More variety in food intake predicts better outcomes [100].The client is also directed to socialize more with other clients. In this example, the client has introverted, obsessive and compulsive traits. These temperament traits are incongruent with treatment expectations.

A temperament-based intervention could have the adult client and clinicians assess their traits and integrate them into the treatment plan. The researchers and developers of TBT-S developed a clinical instrument called, “TBT-S Trait Profile Checklist [98].” It consists of 54 traits common to eating disorders, comorbid illnesses and a subset of traits associated with healthy responses. The traits are identified from research-based temperament studies [101]. The definitions of state versus traits are explained to clients before filling out the self-assessment form. The clients check which traits they have a tendency to express over time. It is not a standardized research instrument. It is a clinical self-assessment for clients to identify some of their traits. The clients are then asked to mark how they have expressed each trait on a continuum between productive and destructive. The Checklist does not frame the traits as good or bad. It directs the client focus on the function of their trait expressions. They are then asked to identify three of their productive traits and three of their more destructively expressed traits. The productive traits become a clinical focus for the client to draw upon to help manage their destructively expressed traits. There are times that clients identify their tendency to express the same trait as both more productive and destructive. This becomes a moment of realization that their same traits can quickly shift in expression under different circumstances.

Simple action-oriented tools from a “TBT-S Toolbox” provide the opportunity for clients to experiment and “try on” actions to help them reroute from destructive symptoms to healthy habits. The tools were developed iteratively by adults clients, researchers, and clinicians. They serve as “transitional actions” that shift direction from destructive to productive responses. The clients are challenged to experiment during the treatment session with the identified tools to explore if they are a “good fit” *with* their traits. The client chooses the action-tools that complement their traits during treatment, setting the agenda to practice the tools when at home or work. The tools that “fit well” are more congruent with their traits, making them easier to use [98]. This sets the stage for potential healthier habit formation.

A “TBT-S Toolbox” example is for clients who have high anixety. One tool is to wear earbuds playing music to counter ED “noise” around meals and times of higher stress. Adult clients report that the music competes with their obsessive anxiety and helps them cognitively cope with the distress when preparing food and after the meal. In a TBT-S approach, the adult client is encouraged to repeatedly practice using tools to replace their destructive “action-tools” of binge eating or purging or excessive exercise.

When clinical intervention “colors” client traits into new habit formations, client-centered productive habits have a greater potential to compete with and replace habitual ED trait-syntonic symptoms. For example, traits such as persistence or determination could be expressed destructively when a client is persistent and determined to purge. The client could utilize the same traits to explore new productive responses that require persistence and determination to complete in a work or school project. As circuits wire together in habit formation, the trait color becomes brighter as the habit strengthens. The trait expression is enhanced through neurocircuit repetitious responses. While one study found that those with AN may not rely on habitual learning any more than those without ED [48], this finding, along with additional research indicates that habits are of central importance for all persons, whether with ED, SEED, recovered from ED, and persons without ED [2, 48, 102, 103]. Habits are fundamental to living. Treatment could mirror how the brain forms habits, through action. The impact of this model could be studied in multiple ways. One could be a random controlled trail comparing the impact of the action-oriented use of TBT-S treatment intervention with treatment as usual. Studies will need to clarify if clients are able to shift their ED actions to trait-syntonic responses. Will they be more motivated to do so? In addition, will traits impact productive actions that can be habitual over time? These are questions that need research to answer.

## Conclusions

This paper offers a metaphoric model of temperament and habit formation to inform how a temperament-based treatment could augment ongoing therapies. It moves beyond former descriptions of temperament traits as constructs and approaches traits in light of newer research, consisting of biological elements that form each person’s traits. The model demonstrates that while temperament traits are constant throughout life, they are malleable in expression.

The model traces how new decisions require a vast amount of neural circuit input from multiple brain areas to collectively inform decisions. If the neural circuit input is accurate, decisions are more likely to be trusted and accurate. If one or more neural circuits are aberrant, then decisions are skewed making it difficult to trust decisions. ED research has found multiple aberrant neural circuits influencing ED behavioural responses. In addition, the model illustrates how traits influence which ED behavioural symptoms a person tends to develop. Repetitious ED behaviours solidify into unhealthy habits. Repetitious productive responses solidify into healthy habits in the *same* way as destructive habits form. Client-centered solutions are formed by the client using their *same* traits and following the *same* neurobiological process that the brain uses when forming habits. The client can draw upon their own traits to discover solutions instead of maintaining their symptoms.

Treatment could capitalize upon the “malleable moments” when new decisions are made and the subsequent new responses that form during therapeutic sessions. These are the moments when neural circuits are formulating and shifting before they become destructive habits. Intervention could help clients to explore trait-syntonic productive responses creating an avenue for productive habits to develop. Repetitive productive actions could compete with symptomatic destructive actions. The habits that persist are the habits most practiced. The model establishes why it is fundamental for treatment to work *with* client temperament to develop healthy trait-syntonic habits to improve the client’s health and quality of life.

## Data Availability

Not applicable.

## Abbreviations

AN: Anorexia nervosa
ED: Eating disorders
SE-AN: Severe and enduring anorexia nervosa
SEED: Severe and enduring eating disorders
DLPFC: Dorsolateral prefrontal cortex, the decision-making area of the brain
TBT-S: Temperament based therapy with support
